# Diagnosing IgE-mediated food allergy: How to apply the latest guidelines in clinical practice

**DOI:** 10.1016/j.jacig.2025.100556

**Published:** 2025-08-18

**Authors:** Carina Venter, Susanne Halken, Alice Toniolo, Anna Nowak-Wegrzyn, Berber Vlieg-Boerstra, Caroline A. Nilsson, David M. Fleischer, Debra de Silva, Domingo Barber, Ekaterina Khaleva, Edward Knol, Jennifer L.P. Protudjer, Laura Morandini, Antonella Muraro, The Global Network of Centres of Excellence for Anaphylaxis & Food Allergy—ANAcare, Pete Smith, Graham Roberts, Helen Brough, Celine Demoulin, Josefine Gradman, Emilia Vassilopoulou, Davide Caimmi, Marcia Podesta, Diana Deleanu, Angel Sanchez, Maria Montserrat Fernandez-Rivas, Pablo Rodriguez del Rio, Anna C. Undeman Asarnoj, Hanneke Oude Elberink, Ronald van Ree, Katherine Anagnostou, Lucy Bilaver, Ruchi S. Gupta

**Affiliations:** a University of Colorado, Aurora, Colo; b Children’s Hospital Colorado, Aurora, Colo; c Hans Christian Andersen Children’s Hospital, Odense, Denmark; d University of Southern Denmark, Odense, Denmark; e Padua University Hospital, Padua, Italy; f Hassenfeld Children’s Hospital, New York, NY; g University of Warmia and Mazury, Olsztyn, Poland; h OLVG Hospital, Amsterdam, The Netherlands; i Rijnstate Hospital, Arnhem, The Netherlands; j Vlieg Dieticians, Arnhem, The Netherlands; k Karolinska Institutet, Stockholm, Sweden; l Sachs’ Children and Youth Hospital, Stockholm, Sweden; m The Evidence Centre, London, United Kingdom; n IMMA, School of Medicine, Universidad San Pablo–CEU (CEU Universities), Madrid, Spain; o University of Southampton, Southampton, United Kingdom; p University Medical Center Utrecht, Utrecht, The Netherlands; q Children’s Hospital Research Institute of Manitoba, Winnipeg, Manitoba, Canada; r The University of Manitoba, Winnipeg, Manitoba, Canada

**Keywords:** Diagnosis, food allergy, oral food challenge, skin prick test, specific IgE

## Abstract

**Background:**

People suspected of food allergy require accurate diagnosis to help manage their condition and get appropriate care. Recent guidelines summarize the latest evidence about diagnosing IgE-mediated food allergy, but they do not describe how to address practical issues when testing. There is a need to translate guideline recommendations into a practical common pathway that all centers dealing with food allergy can use.

**Objective:**

The Global Network of Centres of Excellence for Anaphylaxis & Food Allergy—ANAcare developed a pathway to help clinicians apply the latest diagnostic guidelines and overcome implementation challenges.

**Methods:**

The pathway is based on reviewing guidelines, research and clinical feedback, plus consensus of experts from 13 countries.

**Results:**

We describe practical tips that clinicians can use when taking a detailed clinical history and testing people with suspected IgE-mediated food allergy. Tests for IgE sensitization such as skin prick tests and specific IgE are readily available and inexpensive. However, they only demonstrate sensitization, not clinical allergic disease, so they need to be interpreted in the context of the clinical history. A controlled oral food challenge may also be needed to identify which foods the person is experiencing reactions to and what quantity can be tolerated.

**Conclusions:**

Correct diagnosis is essential to support individualized management. Allergy centers around the world can use our practical tips to help avoid under- and overdiagnosis.

Food allergies involve an abnormal immune-mediated response to food, often a protein.^1^ They can be IgE mediated, non–IgE mediated, or mixed type (Fig 1).^2^ In this report, we focus on IgE-mediated food allergies, hereafter termed *food allergies.*

**Fig 1:**
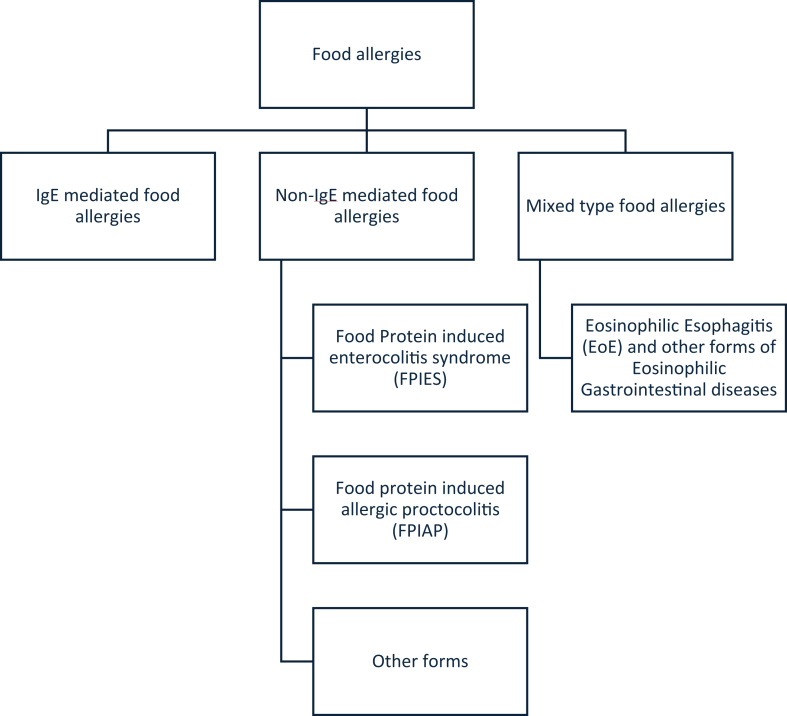
Typology of IgE-mediated, non–IgE-mediated, and mixed-type food allergies.^3^

Food allergy is a major health issue that affects the nutrition, quality of life, and social activities of individuals, their families, and wider communities.^4^ Symptoms can affect any organ system. They vary in their severity, from mild skin symptoms to serious systemic reactions. Symptoms can be upsetting, painful, and even life-threatening.^5^^,^^6^

Food allergies are distinct from other reactions to food that may be based on intolerance and/or that have autoimmune features, such as celiac disease. It is essential to provide prompt and accurate diagnosis for people suspected of food allergy so they can get individualized support about nutrition, foods to avoid, and treatment strategies.

Recent international reviews and guidelines summarize up-to-date evidence about the most effective ways to diagnose and manage food allergies.^1^^,^^7^, ^8^, ^9^, ^10^, ^11^, ^12^, ^13^, ^14^ However, guidelines alone are not sufficient because first, most guidelines focus only on certain elements of the diagnostic process; second, recent guidelines give slightly different recommendations, which may be confusing when trying to apply them in practice; and third, guidelines do not describe how to address practical issues and obstacles when performing diagnostic tests and food challenges.

In order to help patients, guidelines need to be put into practice. The recommendations need to be consistent and interpreted in the same way across settings and regions. Currently, diagnostic guidelines are not being well implemented. There are large variations in the diagnostic approaches used in clinical practice as well as differences in funding, staffing, and processes around the world.^15^ There are also significant regional differences in access to allergy specialists, the use of diagnostic tests, and the ability to perform oral food challenges.^16^

This report is an important step toward addressing these challenges. It is novel in that it combines disparate recommendations from recent guidelines into a common practical pathway that can be used by all centers dealing with food allergy. We showcase how to apply guidelines in practice and provide tips to cope with challenges.

We provide practical steps for health care professionals working with people suspected of food allergy. Our target audience is professionals working in allergy centers, as well as in other hospital roles and in primary care. Not every clinic or setting will be able to undertake all the steps mentioned. We hope that describing a clear pathway concordant with recent guidelines will encourage referrals to other centers where necessary and develop more local capacity and capability over time. Different geographic regions can use this pathway to implement guidelines more consistently and harmonize how diagnostic tests are performed. This may also support more comparable data from different centers to enhance research.

## Methods

In 2024-25, The Global Network of Centres of Excellence for Anaphylaxis & Food Allergy—ANAcare used a consensus approach to develop this guideline-based pathway with novel advice. We set up a working group with representatives from 8 industrialized countries. The working group reviewed guidelines, systematic reviews, and other published evidence. The group used personal communication, surveys, and input at conferences and meetings to gather feedback about good practice, gaps in knowledge, and challenges implementing guidelines. Allergy specialists and centers in 13 countries contributed.

The working group compiled the knowledge into a draft diagnostic pathway and incorporated feedback about the draft from allergy specialists, dieticians, immunologists, researchers, psychologists, primary care teams, and representatives from ANAcare and European Academy of Allergy and Clinical Immunology guidelines groups. We met to review the updated pathway and achieved consensus through discussion. There was consensus across contributors (>90% agreement with each point), so there was no need for a further Delphi process or formal voting.

## Results

### Practical diagnostic pathway

There are 4 key steps in our practical pathway:

1. Clinical history.
2. Sensitization assessment.
3. Oral food challenge.
4. Diagnosis.

In this section, we describe each of these steps, including when to test and how to interpret the test results.

### Step 1: Clinical history

#### Why

**Fig 2:**
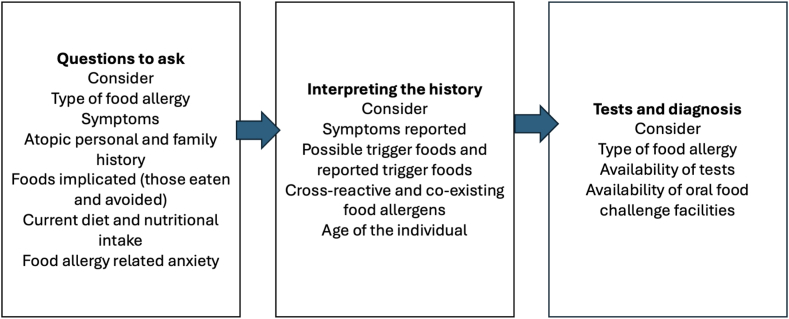
Things to consider in clinical and nutritional history.

People suspected of food allergy can present with a variety of symptoms affecting the skin (urticaria, angioedema, atopic eczema/dermatitis), gastrointestinal (vomiting, abdominal pain, diarrhea), respiratory (rhinorrhea, sneezing, cough, dyspnea), and cardiovascular systems (circulatory collapse). These symptoms can also be nonallergic or caused by other conditions, which need to be considered before diagnosing food allergy. This means it is essential to take a careful clinical history as well as an allergy-focused diet history (Fig 2). The aim is to link allergic symptoms to the foods ingested and to consider the likelihood of food allergy.^17^ Taking a diet history can also help to distinguish primary food allergy versus food allergy that results from cross-reactions with pollens.^18^

Eczema is a special situation because many children with food allergy have eczema. Those with eczema may be suspected of having food allergy, even though eczema is usually not caused by food allergies.^19^ It can be difficult to distinguish between those who have food allergies that affect eczema and those who do not, so guidelines suggest that food allergy should be explored in children with severe persistent eczema and possible food allergy symptoms, especially if combined with other atopic manifestations.

#### How

Box 1 lists practical questions that may be asked during allergy-focused history taking.

Box 1 **Questions to ask during allergy-focused history taking**

**Table:** 

What is the suspected allergen?
Is there any personal history of atopic disease (asthma, eczema, allergic rhinitis)?
Is there any individual and family history of atopic disease (eg, asthma, eczema, allergic rhinitis) or food allergy in parents or siblings?
Are there any cultural and religious factors that affect what the person eats?
Details of any foods that are avoided and the reasons why.
Who raised the concern and suspects food allergy?
What are the presenting and other symptoms that may be associated with food allergy? This may include questions about: • Age at symptom onset. • Speed of onset of symptoms after contact with relevant food. • Duration of symptoms. • Severity of reaction. • Frequency of occurrence. • Setting of reaction (eg, school, home). • Reproducibility of symptoms on repeated exposure, including whether common allergenic foods (eg, milk, eggs, peanuts, tree nuts, soy, wheat, seafood) are usually eaten without symptoms. • What food and how much exposure to it causes a reaction.
Has the patient had any previous treatment, including medication, for presenting symptoms, and what was the patient’s response to treatment?
Any response to eliminating and reintroducing foods.
What is the person’s dietary history? For children/infants, were they breast-fed or formula fed, and what is the age at weaning? What is the mother’s diet if the child is currently being breast-fed?

#### Who

Child and adult allergy specialists, pediatricians, internists, nurses, and dieticians can all be trained to take clinical and diet histories.^20^ In some countries, primary care team members with special interests may be trained. Patients and their families may be asked to keep food diaries to support history taking.

### Step 2: Sensitization

#### Why

If the clinical and dietary history suggests a potential food allergy, the next step is to test for IgE sensitization to the foods. The most common food allergy triggers in industrialized countries are cow’s milk, hen’s egg, peanut, tree nuts, wheat, soy, fish, shellfish (crustaceans, mollusks), and sesame.^2^ Testing is meant to confirm the clinical history rather than being diagnostic alone.

Panel testing (testing a large variety of allergens) should be avoided to prevent overdiagnosis, especially in patients with eczema, who often have high levels of total IgE.^21^ Only foods suspected of causing allergic reactions, chosen on the basis of clinical and dietary history, should be tested to determine sensitization. There is no need to test for food-specific IgE antibodies if the food is tolerated or if the food is not part of the family’s regular diet. This helps prevent unnecessary testing and reduces the risk of false-positive results. High total and specific IgE levels in individuals with eczema may also lead to overdiagnosis.

A positive test result only means that there is sensitization. The test results must always be linked to the patient’s symptoms. An oral food challenge may be performed to determine clinical reactivity if the medical history is unclear.

#### Who

Sensitization testing usually takes place in a hospital allergy center or an allergy clinic with sufficient experience in food allergy. In some areas, general practitioners are trained to perform first-line tests.

#### How

**Fig 3:**
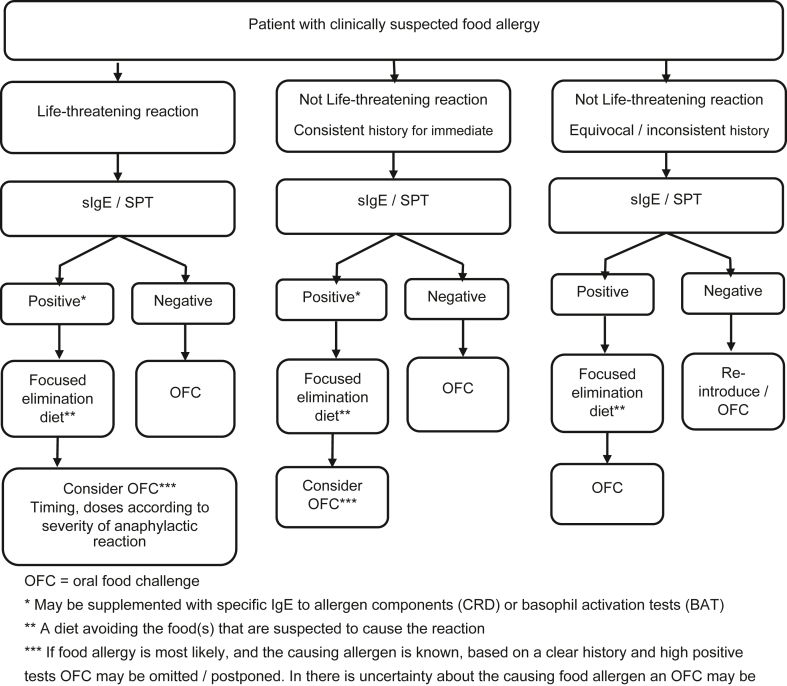
Decision chart for testing to diagnose food allergy.

Table I lists a range of potential tests, and Fig 3 shows when these may be indicated. Box 2 lists tests that are not validated or recommended.^22^^,^^23^

**Table I:** Sensitization tests

Test	Advantages	Disadvantages	Sensitivity	Specificity
SPT, commercial extracts	• Standardized • Fast	• Not available for all foods • Cost • Depends on skilled staff • Difficult to compare over time	• Varies	• Varies; 80-90% to cow’s milk, hen’s egg, peanut, shrimp, hazelnut, cashew, sesame (though can vary between regions)
SPT, prick-to-prick, fresh/frozen foods	• Available for all relevant foods so able to test for culprit food • Inexpensive • Fast	• Not standardized • Most not validated	• May be higher or lower than using commercial extracts	• In some cases low
Specific IgE, whole food allergens	• Standardized • Automated	• Not available for all foods and locations and/or practice settings • Cost	• Varies, but best and moderate (80%) to cow’s milk, hen’s egg, peanut, hazelnut, cashew	• Varies, but best (>80%) to cow’s milk, hen’s egg, egg white, peanut, sesame; <80% for wheat and soy
Specific IgE, individual allergen components (CRD)	• Standardized • May help differentiating between clinical reactions and immunologic cross-reactivity	• Not available for all foods and locations and/or practice settings • Cost	• High (>90%) to cashew Ana o 3 • Moderate to peanut Ara h 2, Ara h 6 • Low for walnut Jug r 1	• High to cashew Ana o 3, peanut Ara h 2 and Ara h 6, hazelnut Cor a 14, walnut Jug r 1 • Moderate to hazelnut Cor a 9
Basophil activation test	• Standardized	• Only validated for peanut and sesame • Not available for all foods and locations and/or practice settings • Cost	• Moderate to peanut (86%) and sesame (89%)	• High to peanut (90%) and sesame (93%)

Data from Santos et al^1^ and Muraro et al.^11^

Box 2 **Tests that are unvalidated or not yet recommended**

**Table:** 

Tests that are not recommended for food allergy testing because of no or insufficient evidence • Allergen-specific IgG and IgG_4_ subclass test. • Antigen leukocyte cellular antibody test. • Electrodermal test. • Hair analysis. • Applied kinesiology. • Iridology. • Gene analysis (eg, detecting polymorphisms). • Vega test. • LEAP Mediator Release test.
Tests that have been used in research settings and may be promising but do not currently have sufficient evidence for use in diagnosing IgE-mediated food allergy in usual clinical practice • Specific IgE to allergen peptides. • Mast cell activation test. • IgG_4_/IgE ratios and allergen/total IgE ratios. • Atopy patch test.

*LEAP,* Learning Early About Peanut Allergy study. Data from Santos et al,^1^ National Institute for Health and Care Excellence (NICE),^22^ and Kelso.^23^

Sensitivity, specificity, and diagnostic accuracy vary considerably between the tests and for different allergens. Access to tests differs by geographic location. Practical points to consider when deciding which tests could be useful include:

• All tests can be positive after someone has tolerated a food. Therefore, it is important not to test foods that the patient tolerates and routinely eats.
• *In vivo* skin prick test (SPT) and *in vitro* allergen-specific IgE in serum for food allergens should be the first tests used to assess IgE sensitization.
• SPTs are cheap and easy to perform. The cost of commercial extracts can limit their use in some regions. For some foods, SPTs with fresh, dried, or frozen food are more sensitive and specific than using commercial extracts, although this varies.^1^
• Tests measuring specific IgE to individual allergens and components/proteins (component-resolved diagnostics [CRD]) can help to differentiate between primary and secondary food allergy but are not available in all allergy centers. Measurement of specific IgE to allergen components, such as Ara h 2 from peanut, Cor a 14 from hazelnut, and Ana o 3 from cashew, may help support a diagnosis, especially in pollen-sensitized people.^15^
• Basophil activation tests can assess allergic responses and may be useful for peanut and sesame, but they are not available in all allergy centers and are more costly than CRD. Therefore, basophil activation tests should be reserved for special cases where SPT, allergen-specific IgE, and CRD are not sufficient.^13^

**Fig 4:**
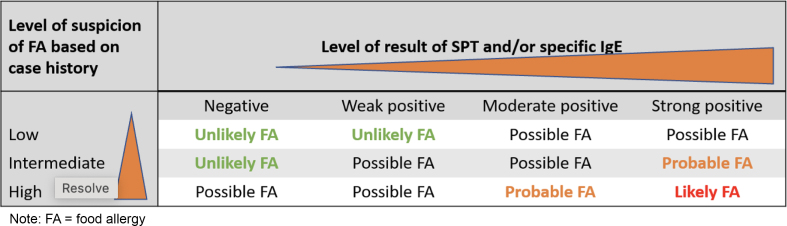
Likelihood of clinical food allergy based on combination of level of clinical history and test results.

In general, the likelihood of food allergy increases the larger the size of the SPT wheal and the higher the level of allergen-specific IgE (Fig 4). Negative results usually have a high specificity and high negative predictive value. However, no test is absolute. All results need to be considered in the context of the clinical history.

Positive predictive cutoff values have been proposed for some allergens, but they are influenced by factors including geographic differences in prevalence, the population, and the test methods and extracts used.^1^ In infants, symptoms of IgE-mediated food allergy may be associated with lower levels of SPT reactivity compared to older children.

### Step 3: Oral food challenge

#### Why

A food allergy will be suspected if a person has an immediate objective allergic reaction after consuming a specific food (usually within 2 hours) and tests suggest IgE sensitization to that food. An oral food challenge may be needed if the connection between a specific food and an allergic reaction is less obvious or if the diagnosis is uncertain. Examples include reactions after complex meals or composite foods, delayed reactions, reactions after ingesting a food previously tolerated, or when cofactors are present such as infections, exercise, and drugs. Even with an anaphylactic reaction, a challenge under controlled conditions may be preferred to ensure a correct diagnosis and a safe diet/treatment if there is doubt about the eliciting food allergen. Challenges may also be performed to test how much of the allergen the person can ingest before experiencing a reaction or whether a person has outgrown the allergy. For example, a patient with a milk allergy and signs of declining IgE/SPT may need to undergo a challenge to determine whether he or she has become tolerant to cow’s milk or to baked or heated forms of milk.

#### Who

In most cases, oral food challenges should be done by health care professionals with experience performing the challenges, interpreting the results, and managing allergic reactions, including anaphylaxis. Most challenges should be done in a setting with access to intensive care equipment. A risk assessment should be carried out about where and how the challenge should be performed.

Oral food challenges are not available in all allergy centers. Where there is a low risk of reaction or a very low risk of serious reaction, sometimes families may perform oral food challenges at home after discussion with their clinician. We do not recommend that this be done routinely because it is difficult to predict the severity of reactions,^1^ and home-based challenges are associated with overdiagnosis. Any positive challenge performed at home should be followed by a clinic-based challenge.

#### How

Challenges involve administering increasing quantities of the allergen to see whether the patient reacts. Clinicians should discuss the goals of the challenge with patients beforehand and provide clear advice and follow-up after the challenge about avoiding or reintroducing foods. Table II lists some of the practical factors to consider when conducting a challenge.

**Table II:** Practical points to consider during oral food challenge

Issue	Potential solutions
Special dietary needs (kosher, halal, vegetarian, vegan)	• Discuss appropriate foods to provide or bring to challenge.
Picky eater	• Discuss likes/dislikes, food and texture aversions, and food preferences before challenge. • Assess eating skills and feeding behaviors. • Be prepared to mix foods with a variety of different options. • Ask caregiver to bring liked and tolerated foods to challenge. • Blenders and mortars are helpful to change food texture to smoother consistency during challenge. • Add crispy texture to foods by adding grain flakes, crackers, or crumbs. • In some cases, guidance from a feeding therapist may be required.
Anxiety	• Child specialist, play therapist, or psychologist may facilitate food consumption and provide support during tests. • Ask caregivers to bring distractions such as toys, games, and movies to occupy child during challenge. • Blinded challenge may help with anxiety for both caregiver and child.

Data from Bird et al.^7^

Challenges can be open or blinded. With single or double-blind challenges, patients do not know whether they are ingesting the allergen as part of a standardized recipe (eg, pudding, cookie, baked product, smoothie). The sensory qualities of the placebo need to be the same as the product with the allergen. Patients should have been able to previously eat all the ingredients in the active preparation and the placebo without symptoms, apart from the allergen itself.^24^^,^^25^ High fat content should be avoided because it may delay symptom development and may lead to severe delayed symptoms.^26^ Some food vehicles, such as wheat, may delay or reduce symptoms and thus may warrant longer observation time.^7^ Food choices may need to be changed for patients with special dietary needs. Double-blind, placebo-controlled oral food challenges are labor intensive but can help identify placebo reactions.^27^ Placebo reactions are more prevalent in children under 1.5 years of age with eczema.^28^

With children, it can help to use their favorite serving dishes and utensils as well as distractions such as toys, books, or homework. In infants and young children, it can work well to mix the allergen dose with a little of something that the child likes and tolerates—for example, a fruit smoothie. For infants or adults with limited chewing ability, the foods used must be pureed and/or thickened.

Patients and/or caregivers may be anxious about consuming food allergens, especially people who have experienced a serious allergic reaction. Symptoms can occur as a result of anxiety rather than the allergen. Psychological support from a professional can help manage anxiety before and during the test and can also help ensure that patients start consuming the food regularly after a negative oral food challenge result.

Various dosing regimens are possible for food challenges. The PRACTALL guidelines for oral food challenges suggest starting at very small doses (3 mg of the allergenic protein) and working through up to 7 stages—3, 10, 30, 100, 300, 1000, and 3000 mg—to reach a cumulative dose of 4.443 g of the allergenic food protein (Table III).^9^^,^^10^ However, in the United States and some other countries, it is more feasible in day-to-day clinical settings to provide a cumulative dose that is equivalent to an age-appropriate serving of food. The cumulative dose is divided into 4 or 6 increasing doses (Table IV).^7^

**Table III:** Common challenge dosing schedule based on PRACTALL guidance

Food protein	Pasteurized cow’s milk (3.3% protein)	Pasteurized whisked hen’s egg (12.8% protein)	Peanut butter (24% protein)	Gluten powder (80% protein)	Soy drink (3.3% protein)
3 mg	91 mg ∼ 0.1 mL	23.4 mg	12.5 mg	3.8 mg	91 mg ∼ 0.1 mL
10 mg	303 mg ∼ 0.3 mL	78.1 mg	41.7 mg	12.5 mg	303 mg ∼ 0.3 mL
30 mg	909 mg ∼ 0.9 mL	234.4 mg	125 mg	37.5 mg	909 mg ∼ 0.9 mL
100 mg	3.03 g ∼ 3 mL	781.3 mg	416.7 mg	125 mg	3.03 g ∼ 3 mL
300 mg	9.09 g ∼ 9.1 mL	2.344 g	1.250 g	375 mg	9.09 g ∼ 9.1 mL
1000 mg	30.3 g ∼ 30.3 mL	7.813 g	4.166 g	1.250 g	30.3 g ∼ 30.3 mL
3000 mg	90.9 g ∼ 90.9 mL	23.438 g	12.500 g	3.750 g	90.9 g ∼ 90.9 mL

Data from Sampson et al.^9^^,^^10^

**Table IV:** Common challenge dosing schedule used in United States based on age-appropriate portion

Allergen	Food	Protein per food portion	Age				
			4-11 months	1-3 years	4-8 years	9-18 years	19+ years
Egg	Egg	6 g/1 large egg	½ to 1 egg	½ to 1 egg	1 egg	1 to 2 eggs	1 to 2 eggs
Grains	Muffin	4 to 6 g per muffin	¼ to ½ piece	½ piece	½ to 1 piece	1 piece	1 piece
Milk	Milk	8 g per 8 fl oz (240 mL)	—	4 to 8 fl oz (120-240 mL)	4 to 8 fl oz (120-240 mL)	8 fl oz (120-240 mL)	8 fl oz (120-240 mL)
Milk	Yogurt (not Greek)	8 g per 8 fl oz (240 mL)	¼ to ½ cup	¼ to ½ cup	½ to 1 cup (125-240 mL)	½ to 1 cup (125-240 mL)	½ to 1 cup (125-240 mL)
Peanut	Peanut (whole)	2 g per 16 peanut piece/halves (∼8 whole peanuts)	—	—	16 peanut pieces/halves (∼8 whole peanuts)	16 peanut pieces/halves (∼8 whole peanuts)	16 peanut pieces/halves (∼8 whole peanuts)
Peanut	Peanut butter	3 g per 1 Tbsp (16 g)	1 rounded Tbsp (∼18 g)	1 to 2 Tbsp (16-32 g)	1 to 2 Tbsp (16-32 g)	2 Tbsp (16-32 g)	2 Tbsp (16-32 g)

Each of these total doses can be divided into 4 (1/12, 1/6, ¼, and ½ of total serving) or 6 doses (1%, 4%, 10%, 20%, 30%, and 35% of total serving). Food values are according to USDA food database (fdc.nal.usda.gov/food-details/746772/nutrients); 249 mL = 8.32 g milk protein and 240 mL = 8 g milk protein. Data from Bird et al.^7^

In Europe, it is more common to use the PRACTALL incremental scale. This is more precise in dosing and may be more suitable for sensitive patients. In a clinical setting, 3 mg of protein seems to be an adequate initial dose for most common food allergens. In most cases, 20- to 30-minute intervals are used between doses, but this may be adjusted according to the patient’s history.^29^ The PRACTALL guidance may not be relevant for certain foods such as fruit or vegetables, where it is not feasible to reach higher protein doses. On the one hand, in these cases, clinicians can use a quantity corresponding to a commonly consumed portion. On the other hand, PRACTALL doses will be too low for protein-rich foods, so extra doses should be added until one portion size is administered.

In some cases, it is useful to consider tolerance starting with the cooked product and then subsequently evaluating the raw food. If spices or aromatic herbs are being tested, clinicians can add a small quantity to a food commonly consumed by the patient, like rice or potatoes, and test the preparation starting from a small amount.

Challenges are usually stopped if clinical reactions are observed or if the last dose is consumed without eliciting clinical symptoms.^11^ The PRACTALL approach suggests grouping signs and symptoms during challenges into 3 categories to aid the decision of when to discontinue or proceed to the next challenge dose:

1. If there are no symptoms, the challenge should continue.
2. If there is a possible reaction, pause and reassess.
3. If there is a definite reaction, stop the challenge.

If observed symptoms are inconclusive, it may be appropriate to consider repeating a challenge dose, delaying the next dose, or stopping the challenge and repeating on another day. Objective symptoms that recur after 3 doses or persist (eg, for 40 minutes) are more likely to indicate a reaction than when such symptoms are transient and not reproducible.^7^ Clinicians need to weigh the risks versus benefits of continuing the challenge (Table V).

**Table V:** Challenge stopping criteria

Organ	Organ category	Scope of symptoms	Comments
Skin	Rash: erythema	• Few areas of faint erythema.∗ • <50% of body surface area.† • Generalized (>50% body surface area).‡	—
	Rash: urticaria	• Limited to perioral region or due to contact.∗ • 1-2 lesions (not perioral or due to contact).† • ≥3 lesions (not perioral or due to contact).‡	Local skin reactions due to contact are excluded (including lip contact with challenge dose).
	Angioedema	• Prominent lip or ear edema.† • Facial edema (and new-onset uvula edema).‡ • Generalized edema.‡	Facial (including periocular) swelling should be prominent and not due to local rubbing or crying. If crying/rubbing causes local swelling, consider delaying next challenge dose to see if other symptoms develop.
	Pruritus	• Scratching (any).∗	Not considered stopping criterion.
Eyes/upper respiratory	Eyes	• Minimal reddening, rubbing of eyes.∗ • Conjunctival hyperemia (without prior rubbing).†	Periocular rubbing or crying commonly cause conjunctival reddening.
	Nasal	• Mild, infrequent rhinitis.∗ • Persistent and significant rhinorrhea, sneezing, and/or rhinitis.†	Mild nasal symptoms are common during challenge and therefore poor indicator of objective reaction.
	Cough	• Intermittent cough associated with throat clearing.∗ • Frequent cough without respiratory compromise.† • Cough with respiratory compromise.‡§	If cough is present without evidence of respiratory compromise (eg, significant tachypnea, decrease in oxygen saturation, use of accessory muscles, wheezing, peak expiratory flow rate decrease of >20% with good technique), consider terminating challenge (which could lead to equivocal result if no other symptoms develop) or adopt watchful waiting (and delay next dose).
	Wheezing	• Any wheeze.‡§	Reduced air entry or added sounds at auscultation may precede wheeze.
	Chest tightness	• Isolated chest tightness.∗ • Chest tightness with decrease in peak flow of ≥20% (with good technique).‡§	Chest tightness is subjective and should not trigger challenge/stop in isolation (but may prompt extending dosing interval). If peak flow is being assessed, then decrease of ≥20% from baseline (assuming satisfactory technique) can be considered stopping criterion.
Oropharyngeal	Oral cavity	• Itchy mouth.∗	—
	Throat/laryngeal	• Itchy throat, intermittent throat clearing.∗ • Persistent throat tightness and/or pain.† • Nontransient. hoarseness/stridor.‡	Subtle vocal changes are presumably due to mild laryngeal edema and should therefore trigger challenge to be stopped if not transient.
Gastrointestinal	Abdominal discomfort	• Nausea (any severity or frequency).∗ • Mild abdominal pain.† • Persistent nondistractable abdominal pain.† • Pain (usually accompanied by decrease in activity level in children).† • Persistent severe abdominal pain.†	Abdominal pain is subjective and should not trigger challenge or stopping in isolation. Persistent severe abdominal pain would normally be accompanied by other symptoms. When this is present, further doses should be deferred to allow additional time for other symptoms to evolve.
	Vomiting	• Vomit due to gag or taste aversion.∗ • 1+ episode (where investigator considers this due to allergic reaction).‡	Vomiting occurring during or shortly after dose is likely to be due to gag or taste aversion. If other symptoms subsequently develop, reconsider whether episode was nonallergic in origin.
	Diarrhea	• 1 episode.† • 2+ episodes.‡	—

Includes 1 severe or 2 moderate symptoms from 2 distinct organ categories.^9^

∗ Viewed as mild during challenge.

† Viewed as moderate within scope of symptoms.

‡ More severe symptoms.

§ Manage as anaphylaxis.

Signs of an IgE-mediated allergic reaction during challenges often include the skin (urticaria, angioedema). They may also include the following systems: cutaneous (urticaria, angioedema, flushing, scratching), gastrointestinal (dysphagia, vomiting, abdominal cramping, diarrhea), respiratory (sneezing, rhinorrhea, coughing, wheezing, stridor, dysphonia, aphonia), cardiovascular (pallor, hypotension, loss of consciousness), and behavioral (change in activity/behavior, especially in young children and infants).

Immediate reactions usually appear within 2 hours of the last food intake, so patients should be observed for at least 1 to 2 hours for an immediate-type reaction.

A food challenge result will be regarded as negative when the patient tolerated the food. This usually means that the amount of food protein ingested without symptoms is equivalent to a normal serving of the food prepared in the usual manner,^30^ except when “low dose” threshold challenges are performed and the target dose is much lower than a usual serving size. These threshold challenges are performed to identify the allergen dose that the patient is reacting to rather than to diagnose food allergy.

After a negative challenge result, the patient should be advised to avoid the food for the rest of the day in case a rare, delayed reaction occurs. The patient/family should be advised to watch for signs of reactivity as the food is reintroduced into the diet in the days and weeks after the challenge and told to contact the clinic if there are delayed reactions or any reactivity.^7^^,^^11^

After a positive challenge result, where the patient reacted to the food, the duration of observation depends on clinical judgment. Patients are often observed for 2 to 4 hours after symptoms end. Timing of observation after an anaphylactic reaction should follow usual local recommendations for anaphylaxis.^7^

All patients should have an emergency treatment plan and medications available when they are discharged after a positive or inconclusive oral food challenge.

There is scope to standardize how the outcomes of diagnostic testing are reported to support using real-life data for ongoing research. For example, the Food Allergy Severity Score can be used when reporting the result of food challenges.^31^

### Step 4: Diagnosis

#### Who

Clinicians need to combine the results of the clinical history, tests for sensitization, and oral food challenges, when used, to form a diagnosis. This is usually done in a specialist allergy center or clinic.

#### How

Clinicians should contemplate differential diagnoses when considering a diagnosis of IgE-mediated food allergy (Table VI). When patients are diagnosed with food allergy, in most cases they should be advised about avoiding the food, carrying adrenaline (epinephrine), interpreting precautionary allergen labeling, and managing worry or anxiety. They should work with their clinician to develop an emergency treatment plan in the event of an accidental exposure or reaction. In some individual cases with mild reactions to high doses, ingesting products with precautionary allergen labels, small amounts of the allergenic food, or, for milk or egg allergy, even baked forms, may be introduced into the diet.^30^

**Table VI:** Examples of differential diagnosis of food reactions and non–IgE-mediated food allergies

Mechanism	Examples
Immunologic	Celiac disease, inflammatory bowel syndrome
Metabolic	Lactose intolerance, galactosemia, FODMAP intolerance, irritable bowel syndrome
Pharmacologic	High histamine-containing foods (aged cheese, fermented meat, fish, sauerkraut) and histamine-releasing foods (strawberry, papaya, wine, kiwi, pineapple), tyramine (aged cheese, pickled fish), caffeine, theobromine (chocolate), phenylethylamine (chocolate), α-solanine (potatoes), TRPV1 and TRPA1 agonists (spices, capsaicin, allicin in garlic and onion, ginger, wasabi, horseradish, pepper), MSG, alcohol, serotonin (tomato, banana), tryptamine (tomato, plum)
Toxic	Infectious gastritis/enteritis and histamine intoxication (scombroid fish poisoning, poisoning from other types of fish or cheese)
Other	(Infectious) gastroenteritis, infection-triggered urticaria, idiopathic urticaria, stress/anxiety, psychogenic reactions, gastroesophageal reflux, unhealthy or unbalanced diet, mast cell disorders
Non–IgE-mediated food allergy	Food protein–induced enterocolitis syndrome, food protein–induced proctocolitis, other presentations of non–IgE-mediated food allergies (mild-to-moderate non–IgE-mediated food allergies, gastroesophageal reflux, other skin and gut symptoms, eosinophilic esophagitis, other presentations of gastrointestinal eosinophilic diseases)

*FODMAP,* Fermentable oligosaccharides, disaccharides, monosaccharides, and polyols; *MSG,* monosodium glutamate.

If patients are not diagnosed with food allergy, they should be advised to consume the food regularly (Table VII). A number of studies suggest that despite this type of advice, up to a third of patients do not introduce the food allergen at home, possibly as a result of food aversion and/or fear of reactions.^32^, ^33^, ^34^

**Table VII:** Suggestions for how to introduce foods if food allergy is not diagnosed

Allergen	Options
Peanut and tree nuts	Peanut/tree nuts contained in nut flour, puffs, breakfast cereals, candies; flours and butters mixed into fruit purees, yogurt, oats, smoothies, meatballs, muffins, pasta sauce, soups, stews
Milk	Milk (fresh/powdered), yogurt, ice cream, cheese (raw or in baked foods)
Egg	Egg in baked foods, meringue toppings and bread/cookies, boiled/poached/fried egg, pancakes

## Discussion

Health care professionals working in allergy centers or clinics and linked settings can use the steps we set out in this report to diagnose people suspected of food allergy. The pathway translates guidelines into practical steps and includes tips to help clinicians do the day-to-day work. If all centers used this approach, people across the world would have access to more harmonized and consistent diagnosis and follow-up care. We recognize that there are differences in access to tests and resources. However, specific IgE tests are easily accessible in most clinical laboratories and could be used and interpreted more consistently.

Researchers continue to work on gaps in existing knowledge, such as the following:

• The predictive value, sensitivity, and specificity of the clinical history in different types of food allergies.
• The value of specific IgE to allergen peptides, mast cell activation test, IgG_4_/IgE ratios, and allergen/total IgE ratios.
• Which type of oral food challenge is most effective for each type of food allergy, including open versus double-blind tests.^35^^,^^36^
• The interval between each dose in oral food challenges and whether this differs between foods or types of reactions.^29^

Diagnosing food allergy is only the first part of the journey. It is essential that people diagnosed with food allergies receive support with long-term management. This includes an individualized management plan, emergency action plan, advice about avoiding or gradually reintroducing food triggers, referral to a dietician for support with nutrition, prescription of adrenaline self-administration devices, discussion of immunotherapy or other treatment options, psychosocial support, and information and support for family caregivers. Assessing the impact of food allergy on quality of life can be an important step in the diagnostic process, acknowledging the wider determinants of health. There are validated questionnaires available to help clinicians screen for the impact of food allergy on quality of life. This can also help identify patients who may benefit from counseling or other support.^26^ The physical consequences of food allergy can be acute and serious but may occur sporadically. However, the psychological impacts of food allergy are ongoing because people may be anxious about having a reaction.^37^^,^^38^ Therefore, once people receive a diagnosis it is essential to put support in place for the affected people and their caregivers.

**Key messages**

• Correct diagnosis is essential to support individualized management for people suspected of food allergy. Allergy centers use different approaches, which may lead to inconsistent outcomes.
• The pathway described in this report helps centers overcome practical obstacles when testing.
• Clinicians should take a detailed clinical and dietary history, followed by tests to demonstrate IgE sensitization and oral food challenges if needed to identify trigger foods.

## Disclosure statement

The Global Network of Centres of Excellence for Anaphylaxis & Food Allergy—ANAcare supported experts to attend conferences and meetings where this document was discussed and edited.

Disclosure of potential conflict of interest: C. Venter declares institutional grant from Reckitt; and speaker fees from Danone, Reckitt, Nestlé Nutrition Institute, and Abbott Nutrition. S. Halken declares consultation fees from ALK-Abelló, Nestlé Purino, Abigo, Meadjohnson; and data monitoring committee for Stallergenes. A. Nowak-Wegrzyn declares institutional grants from Alladapt Immunotherapeutics, DBV Technologes, Siolta Therapeutics, and Regeneron; speaking fees from Nestlé, Danone, and Thermo Fisher Scientific; consulting fees from Aquestive; royalties from UpToDate; and service as associate editor for *Annals of Allergy, Asthma & Immunology,* chair of the ABAI board of directors, director of the AAAAI board, and chair of the medical advisory board of the International FPIES Association. B. Vlieg-Boerstra declares institutional grants from Nutricia and OLVG Research; and consulting or speaker’s fees from Nestlé, Vini Mini, Colzaco, and Nutricia. D. M. Fleischer declares grants from DBV Technologies and ARS Pharmaceuticals; royalties from UpToDate; consultancy fees from Aquestive, ARS Pharmaceuticals, Bryn Pharma, DBV Technologies, Genentech, and Nasus Pharma; speaker for Genentech; stock options from Grow Happy; and service as unpaid member of the medical advisory board for the Food Allergy & Anaphylaxis Connection Team (FAACT) and the Medical Advisory Council for the National Peanut Board. D. de Silva declares an institutional grant from ANAcare toward report editing. D. Barber declares lecture fees from Diater Labs. E. Knol declares speaker and/or consultant fees from Sanofi, Thermo Fisher Scientific, and GSK. J. L. P. Protudjer declares consulting fees from Ajonomoto Cambrooke, Novartis, Nutricia, and ALK Abelló; and service as section head of Allied Health, colead of Research Pillar for the Canadian Society of Allergy and Clinical Immunology, and member of the steering committee for Canada’s National Food Allergy Action Plan. A. Muraro declares speaker fees from Aimmune, DBV Technologies, Nestlé Health Science, and Nestlé Purina; and advisory boards for Aimmune, Sanofi, DBV Technologies, Novartis, Regeneron, and Viatris. The rest of the authors declare that they have no relevant conflicts of interest.
